# The Methanol Dehydrogenase Gene, *mxaF*, as a Functional and Phylogenetic Marker for Proteobacterial Methanotrophs in Natural Environments

**DOI:** 10.1371/journal.pone.0056993

**Published:** 2013-02-22

**Authors:** Evan Lau, Meredith C. Fisher, Paul A. Steudler, Colleen M. Cavanaugh

**Affiliations:** 1 Department of Natural Sciences and Mathematics, West Liberty University, West Liberty, West Virginia, United States of America; 2 Department of Organismic and Evolutionary Biology, Harvard University, Cambridge, Massachusetts, United States of America; 3 The Ecosystems Center, Marine Biological Laboratory, Woods Hole, Massachusetts, United States of America; University of Delaware, United States of America

## Abstract

The *mxaF* gene, coding for the large (α) subunit of methanol dehydrogenase, is highly conserved among distantly related methylotrophic species in the Alpha-, Beta- and Gammaproteobacteria. It is ubiquitous in methanotrophs, in contrast to other methanotroph-specific genes such as the *pmoA* and *mmoX* genes, which are absent in some methanotrophic proteobacterial genera. This study examined the potential for using the *mxaF* gene as a functional and phylogenetic marker for methanotrophs. *mxaF* and 16S rRNA gene phylogenies were constructed based on over 100 database sequences of known proteobacterial methanotrophs and other methylotrophs to assess their evolutionary histories. Topology tests revealed that *mxaF* and 16S rDNA genes of methanotrophs do not show congruent evolutionary histories, with incongruencies in methanotrophic taxa in the Methylococcaceae, Methylocystaceae, and Beijerinckiacea. However, known methanotrophs generally formed coherent clades based on *mxaF* gene sequences, allowing for phylogenetic discrimination of major taxa. This feature highlights the *mxaF* gene’s usefulness as a biomarker in studying the molecular diversity of proteobacterial methanotrophs in nature. To verify this, PCR-directed assays targeting this gene were used to detect novel methanotrophs from diverse environments including soil, peatland, hydrothermal vent mussel tissues, and methanotroph isolates. The placement of the majority of environmental *mxaF* gene sequences in distinct methanotroph-specific clades (Methylocystaceae and Methylococcaceae) detected in this study supports the use of *mxaF* as a biomarker for methanotrophic proteobacteria.

## Introduction

Atmospheric methane contributes to ∼20% of the total radiative forcing by long-lived greenhouse gases globally. Due to the relatively short lifetime of atmospheric methane (∼9 yrs) compared to CO_2_, reductions in atmospheric methane flux would have an immediate impact on global warming [1]. Microbial methane oxidation is the only major biological sink of methane [2]. With the exception of members of the phylum Verrucomicrobia [3], and the yet to be cultured anaerobic methane-oxidizers (ANME) [4], [5], and denitrifying methanotrophs of the ‘NC10’ phylum, aerobic proteobacterial methanotrophs are the only currently known groups of microorganisms capable of oxidizing methane as their sole carbon source [6], [7], [8], thus reducing atmospheric methane flux.

Aerobic proteobacterial methanotrophs are unique among the larger group of methylotrophic bacteria (which are able to utilize C_1_ or one-carbon compounds) in that they oxidize methane to methanol, before subsequent metabolic reactions that are shared with other methylotrophs. In contrast, non-methanotrophic methylotrophs are unable to utilize methane, but can grow on other C_1_ compounds (e.g., methanol, methylated amines, formate, or formamide) [9], [10]. The vast majority of known aerobic methanotrophs belong to the Proteobacteria. Known exceptions include the phylum Verrucomicrobia, whose pathways for methanotrophy are still poorly understood [3], [11], [12], and enrichments of the uncultured methanotrophs such as *Candidatus* ‘Methylomirabilis oxyfera’ affiliated with the ‘NC10’ phylum, which appear to be incapable of oxidizing methane at low (≤8%) oxygen levels in the laboratory [7], [8], [13]. Proteobacterial methanotrophs are placed in the families Methylococcaceae in the Gammaproteobacteria, and Methylocystaceae and Beijerinckiaceae in the Alphaproteobacteria [14], [15].

The similarities in physiological characteristics, and the generally highly conserved nature of the 16S rRNA and functional gene sequences of proteobacterial methanotrophs, have enabled the use of targeted PCR primers for gene amplification to describe methanotroph diversity [15], [16]. However, the 16S rRNA gene is non-protein coding (i.e., not linked directly to methanotroph physiology), and therefore does not directly determine function. For example, it cannot be determined whether 16S rDNA environmental sequences placed close to, but outside of, known monophyletic methanotroph clades are indeed methanotrophic [17], [18]. In comparison, functional genes for enzymes found in methanotroph metabolic pathways are directly related to physiology [19]. The initial step of methane oxidation to methanol by proteobacterial methanotrophs is mediated by particulate and/or soluble methane monooxygenases (MMOs) [15], [20]. Genes in this step, such as *pmoA* [encoding a subunit of the particulate methane monooxygenase (pMMO)] and *mmoX* [encoding a subunit of the soluble methane monooxygenase (sMMO)] have been used for describing methanotroph diversity [17]. However, the *pmoA* gene is not present in the genera *Methylocella* and *Methyloferula* [21], [22], [23], and the *mmoX* gene is present only in a few strains of methanotrophs [15], [24]. Hence, the use of both *pmoA* and *mmoX* genes in PCR-based studies results in the underestimation of methanotroph diversity and suggests that an alternative is needed.

The *mxaF* gene was first proposed as a functional gene probe for methylotrophs by McDonald and Murrell [19]. Methane oxidation gene cluster A (*mxaF*) encodes the α-subunit of the enzyme methanol dehydrogenase (MDH), an enzyme containing a pyrroloquinoline quinone (PQQ) cofactor that oxidizes methanol to formaldehyde in the second step of the methane oxidation pathway, following oxidation of methane to methanol [20], [25], [26], [27], [28]. Methanol dehydrogenase is common to all methanotrophs in the Proteobacteria [15], [19], but is absent in members of the phylum Verrucomicrobia, which possess a homolog of the *mxaF* gene, called *xoxF* gene, a gene of unknown function [29], [30], [31], [32]. Sequences of the *mxaF* gene are highly conserved in methylotrophic species in the Alpha-, Beta- and Gammaproteobacteria, reflecting 16S rDNA phylogeny [19], [32], [33]. In studies thus far based on the *mxaF* gene, a limited number of representative methanotrophs (<15 taxa) have been placed in generally distinct and coherent clades separate from the other methylotrophs [15], [19], [24], [32]. These studies indicate the *mxaF* gene can be a useful phylogenetic marker for the classification of methanotrophs.

However, the extent to which *mxaF* gene sequences of all known proteobacterial methanotrophs can be placed in separate distinct phylogenetic clades has yet to be determined. This may affect its accuracy as a reliable functional biomarker and potentially lead to incorrect inferences when determining phylogenetic relationships, notably if horizontal gene transfer has occurred across these taxa. To date, no extensive phylogenetic comparisons of proteobacterial methylotroph (including methanotroph) *mxaF* gene sequences have been conducted. In the only study of *mxaF* phylogeny of the family Methylocystaceae (genera *Methylosinus* and *Methylocystis*), representative *mxaF* genes sequences from this family clustered within a distinct clade, but did not separate according to genus-specific subclades, suggesting that horizontal transfer of this gene may have occurred across this family. However the study did not indicate the extent of horizontal gene transfer in other proteobacterial methanotrophs, and which taxa were involved [17]. Previous studies of *mxaF* revealed that it is related to quinone alcohol dehydrogenases (ADHs), which utilize a variety of primary and secondary alcohols, but not methanol, as substrates [34], and that *mxaF* is related to *xoxF* [32].

Here, we examined whether the *mxaF* and 16S rRNA gene phylogenies of methanotrophs from the GenBank [35] database reflect congruent evolutionary histories. Though *Candidatus* M. oxyfera (and members of NC10 phylum) possesses an *mxaF*-like gene, it is placed outside of known proteobacterial *mxaF* gene clusters (data not shown). *Candidatus* M. oxyfera’s genome lacks any known PQQ-biosynthesis pathways and thus may not be able to oxidize methanol on its own [8]. Hence it was not included in our analyses.

This study addresses the following questions, (a) Does the extensive *mxaF* gene phylogeny discriminate between methanotrophic and other methylotrophic sequences available in genetic databases? (b) Are the *mxaF* and16S rRNA gene phylogenies congruent for methanotrophs in the families Methylocystaceae, Methylococcaceae, and Beijerinkiaceae? (c) Can we tell whether *mxaF* genes retrieved from the environment belong to methanotrophs or methylotrophs? These questions are addressed using phylogenetic analyses involving sequences from published databases, as well as *mxaF* sequences obtained in this study from surveys of diverse environments (forest soils, peat and *Sphagnum* moss, and symbiont-hosting mussel gills) and from methanotroph cultures. Overall, these analyses advance the study of methanotroph diversity by showing that the *mxaF* gene consistently places methanotroph sequences in resolved phylogenetic clusters for all known members of the families Methylocystaceae and Methylococcaceae, and has the potential to elucidate the roles methanotrophs play in natural environments.

## Materials and Methods

### Cultures

Two *mxaF* gene sequences were determined in this study for cultured species of the Methylococcaceae, *Methylomonas rubra* and *Methylobacter luteus*, provided by J. Semrau and R. Knowles, respectively. Positive control cultures used in this study included two members of the Methylocystaceae and a member of the Methylococcaceae for which *mxaF* gene sequences are already available: *Methylosinus trichosporium* OB3b, *Methylocystis parvus* OBBP, *Methylomicrobium album* BG8, respectively.

### Collections and Site Descriptions

Samples for DNA extraction or bacterial isolation were collected from four distinct habitats for DNA extraction or bacterial isolation: (a) Four soil samples, weighing ∼15 g, from the organic horizon of long-term nitrogen-amended and control pine and hardwood forest soils of the Harvard Forest Long-Term Ecological Research (LTER) site, previously shown to oxidize methane at atmospheric levels, in Petersham, Massachusetts (42°30′N, 72°10′W) in 2004 [18], [36], (b) *Sphagnum recurvum* moss and peat from Crystal Bog (Vilas county, Wisconsin), a 7 ha poor fen enclosing a 2.5 m deep, 0.54 ha, dystrophic lake on the North Temperate Lakes LTER site in 2005 (46°00′30″N 89°36′30″W) [37], (c) two species of methanotroph-hosting mussels (n = 3 per site) from the Mid-Atlantic Ridge (MAR) deep-sea vent sites sampled in 2003 using DSV Alvin: *Bathymodiolus azoricus* from Lucky Strike (LS; 37°17′N, 32°16′W; 1693 m deep) and Rainbow (RB; 36°13′N, 33°54′W, 2300 m deep), and *Bathymodiolus puteoserpentis* from Logatchev (LO; 14°45′N, 44°58′W; 3027 m deep) [38], [39], and (d) the Halls Brook Holding Area (HBHA), an artificial lake in the Aberjona Watershed, sampled in 2004, near Boston, MA, which becomes stratified during the summer months, whereby the bottom depths become anoxic [40]. No specific permissions were required for collecting samples in these locations because samples did not involve endangered or protected species, Harvard Forest is owned by Harvard University and permission is granted to research employees, and Crystal Bog and Halls Brook Holding Area are located on public land.

### Methanotroph Isolation, DNA Extraction, and Purification

Methanotrophic HBHA isolate 1 and HBHA isolate 2 were isolated from water collected at the oxic-anoxic interface at HBHA (∼1 m depth) using sterile Tygon® tubing connected to a peristaltic pump, and injected into sterile flasks containing Nitrate Mineral Salts (NMS) minimal medium [41], under 90∶10 air:methane headspace, and incubated with shaking at room temperature. Both isolates failed to grow in the absence of methane and oxygen. Purified DNA from methanotroph cultures was obtained using the Wizard® Genomic DNA Purification Kit (Promega Inc.). DNA from endosymbiont-containing mussel gill tissue and forest soils was obtained as previously described [18], [38]. DNA was extracted from HBHA isolates, peat and *Sphagnum* moss following the method as previously described [18].

### Primer Design, PCR Amplification, Cloning, and Sequencing

Methylotroph *mxaF*-specific PCR primers F1003 and R1561 ( [33]; Table 1) sequences were verified through BLAST [42] to determine their specificity to proteobacterial methanotrophs in the GenBank [35] database, and used to amplify partial *mxaF* gene sequences from soils, peat, *Sphagnum* moss, methanotrophic HBHA isolates 1 and 2, and control methanotroph cultures. The amplified region encompasses three amino acid residues (out of eight) in the MDH active sites – Asn-261, Asp-303 and Arg-331– based on the amino acid sequence of *Methylobacterium extorquens* MDH [26]. BLAST searches of the primer pair F1003 and R1561 retrieved only proteobacterial *mxaF* genes, but not that of *Candidatus* ‘Methylomirabilis oxyfera.’

**Table 1:** PCR Primers sets used in this study.

Name	Sequence (5′ → 3′)	Reference
F1003	GCGGCACCAACTGGGGCTGGT	33
R1561	GGGAGCCCTCCATGCTGCCC	33
F1003degen	GGNCANACYTGGGGNTGGT	This study
R1561degen	GGGARCCNTTYATGCTNCCN	This study

Additionally, degenerate primers F1003degen and R1561degen (Table 1) were created after comparing amino acid sequences of MDH retrieved from the GenBank database and identifying degenerate base positions where more than one nucleotide codon specified the same amino acid residue. The degenerate primers were used to PCR amplify *mxaF* genes from DNA extracts of mussel tissues, as primers F1003 and R1561 failed to PCR amplify *mxaF* genes from the same DNA extracts. The same primers were also used to PCR amplify *mxaF* genes from *Sphagnum* moss in order to verify these primers also PCR amplify *mxaF* sequences outside of the Methylococcaceae. All PCR reaction mixtures contained 1× PCR buffer (Qiagen Inc.), 2.5 mM MgCl_2_, 300 mM final concentration of each dNTP, a 1.0 µM final concentration of each primer, 1.0 U of Taq polymerase, and approximately 300–800 ng of template DNA in a final volume of 25 µl. PCR conditions were: denaturation at 94°C for 45 sec, primer annealing at 60°C for 1 min, extension at 72°C for 1.5 min for 30 cycles, and a final 10 min extension at 72°C. PCR products of the expected size (∼550 bp for *mxaF* gene) were purified, cloned, and sequenced (>3 clones per sample).

### Cloning and Sequencing

PCR products of the expected size (∼550 bp for *mxaF*) were purified (QIAquick PCR Purification Kit, Qiagen Inc.), cloned (pCR®2.1-TOPO vector from the TOPO TA Cloning Kit, Invitrogen Corp.) using chemical method on TOP 10 competent cells, and plated on LB agar plates containing 50 µg ml^−1^ kanamycin, and 40 mg ml^−1^ X-Gal (5-bromo-4-chloro-3-indolyl-β-D-Galactopyranoside). Colonies were screened for inserts using the respective primers via PCR. Plasmid DNA was isolated from positive clones using the QIAprep^â^ Spin MiniPrep Kit (Qiagen Inc.). Sequencing reactions were performed using the ABI PRISM® Big Dye Terminator Cycle Sequencing kit (version 3.1, Applied Biosystems®) and an ABI model 3100 automated sequencer (Applied Biosystems®) according to the manufacturer’s instructions. Recombinants were sequenced in both directions using M13 forward and reverse primers. For environmental DNA extracts, at least five clones were sequenced from each PCR reaction. For *Bathymodiolus* mussels, three clones each were sequenced from frozen gill tissue of three *B. azoricus* mussels from LS, three *B. azoricus* mussels from RB, and three *B. puteoserpentis* mussels from LO, for a total of 27 sequences.

### Phylogenetic Analyses of Methylotrophic Bacterial Sequences from GenBank Database

The ADH gene and/or 16S rRNA gene sequences of *Solibacter usitatus* Ellin 6076 (GenBank Accession no. NC_008536/CP000473, Phylum Acidobacteria) were used as outgroup in all phylogenetic analyses. *mxaF* gene sequences were aligned using ClustalW2 (http://www.ebi.ac.uk/Tools/msa/clustalw2/) and manually inspected in MacClade 4.08 (Sinauer Associates Inc., Sunderland, MA). Phylogenetic reconstruction was implemented using PAUP 4.0B10 [43] and Geneious 4.85 (http://www.geneious.com/) with PAUP plug-in. Unless stated otherwise, statistical support for all trees were obtained from 1,000 bootstrap replicates under the same initial settings (only bootstrap values >50% are reported). Pairwise base comparisons of *mxaF* nucleotide sequences within and between phylogenetic groups were determined using ClustalW2 and reported as % identity values.

#### (a) Congruency tests between mxaF and 16S rRNA gene tree topologies

The program Modeltest [44] was used to determine the bestfit substitution model under Maximum Likelihood (ML) analysis (from PAUP software) for each dataset, and searched heuristically for the best model of nucleic acid sequence evolution that best fits our data. The topology of the best tree from ML analyses of each dataset was saved and then enforced as a topological constraint during subsequent paired ML phylogenetic analyses for each dataset. One-tailed Templeton and Shimodaira-Hasegawa (SH) tests were used to compare the constrained and unconstrained topologies using reestimated log likelihoods (RELL) simulation [45], [46] and full optimization distributions using 10,000 bootstrap replicates under the likelihood tree scores menu. Uncorrected and Bonferroni-corrected *P*-values were reported for one-tailed Templeton and SH tests [45]. Confidence intervals were determined for the null hypotheses: the unconstrained and constrained ML tree of each dataset tested have significantly different likelihood scores. The data consisted of nineteen methanotroph taxa spanning three families (Methylocystaceae, Beijerinckiaceae, and Methylococcaceae) and one outgroup for which both genes were available: (a) seven members of the Methylocystaceae – *Methylosinus trichosporium* BF1, *M. trichosporium* O19/1, *M. trichosporium* KS21, *Methylosinus sporium* F10/1b, *Methylocystis echinoides* IMET10491, *Methylocystis* sp. IMET10489, and *Methylocystis* sp. IMET10484, (b) four members of the methanotrophic Beijerinckiaceae – *Methylocella silvestris, M. tundra, M. palustris,* and *Methylocapsa acidiphila*, and (c) eight members of the Methylococcaceae – *Methylococcus capsulatus* Bath, *Methylocaldum* sp. E10a, *Methylomonas methanica*, *M. album*, *M. rubra*, *Methylobacter luteus*, *Methylobacter* sp. 5FB, and *Methylomicrobium* sp. For the Methylococcaceae, there are insufficient *mxaF* sequences in GenBank, hence the *mxaF* of *Methylomicrobium album* str. BG8 (GenBank Accession no. L33682) was matched to the 16S rRNA gene sequence of *Methylomicrobium* sp. (GenBank Accession no. D89279). Separate ML phylogenetic analyses were conducted on three datasets: (a) *mxaF* gene sequences (∼513 bp) of these methanotrophs, (b) the corresponding 16S rRNA gene sequences (∼1471 bp) of the same taxa and (c) the concatenated *mxaF* and 16S rRNA gene sequences (∼1984 bp).

#### (b) Phylogenetic analyses of cultured methanotrophs and other related methylotrophs

Maximum parsimony (MP) phylogenies of known methylotrophs (including methanotrophs), and related *xoxF* PQQ-linked dehydrogenase genes, which have recently been described [32] and were included to verify their phylogenetic placement relative to *mxaF* genes, were constructed based on nucleotide and on inferred amino acid sequences (n = 145) available in Genbank [35]. Alignments for *mxaF* nucleotide sequences included sequences with and without third nucleotide positions. Phylogenetic analyses (MP) implemented included alignments for *mxaF* nucleotide sequences with and without third nucleotides to assess third codon position bias) and inferred *mxaF* amino acid sequences (513, 342, and 171 characters, respectively) for 145 taxa. The resulting tree was obtained via random stepwise addition of sequences, as consensus of 9 saved, most parsimonious trees, obtained from a heuristic search.

### Phylogenetic Analyses of Environmental mxaF Gene Sequences

All environmental *mxaF* nucleotide sequences detected in this study were verified through BLAST searches, and *mxaF* nucleotide sequences of their closest relatives (identified through BLAST searches) were included in the assembly. The final alignments for *mxaF* nucleotide sequences, *mxaF* sequences without third nucleotides and inferred *mxaF* amino acid sequences comprised 513, 342, and 171 characters, respectively, for 85 taxa. Maximum parsimony (MP) methods (implemented as described above) were used to generate phylogenetic trees from the nucleotide alignments. The resulting tree was obtained as consensus of 4 saved, most parsimonious trees obtained from a heuristic search, with random stepwise addition of sequences.

### Nucleotide Sequence Accession Numbers

The *mxaF* gene nucleotide and inferred amino acid sequences of *Methylomonas rubra* and *Methylobacter luteus*, HBHA isolates 1 and 2, and uncultured clones have been deposited in GenBank, under accession numbers JX312966-JX313018.

## Results

### Congruency Tests

**Figure 1:**
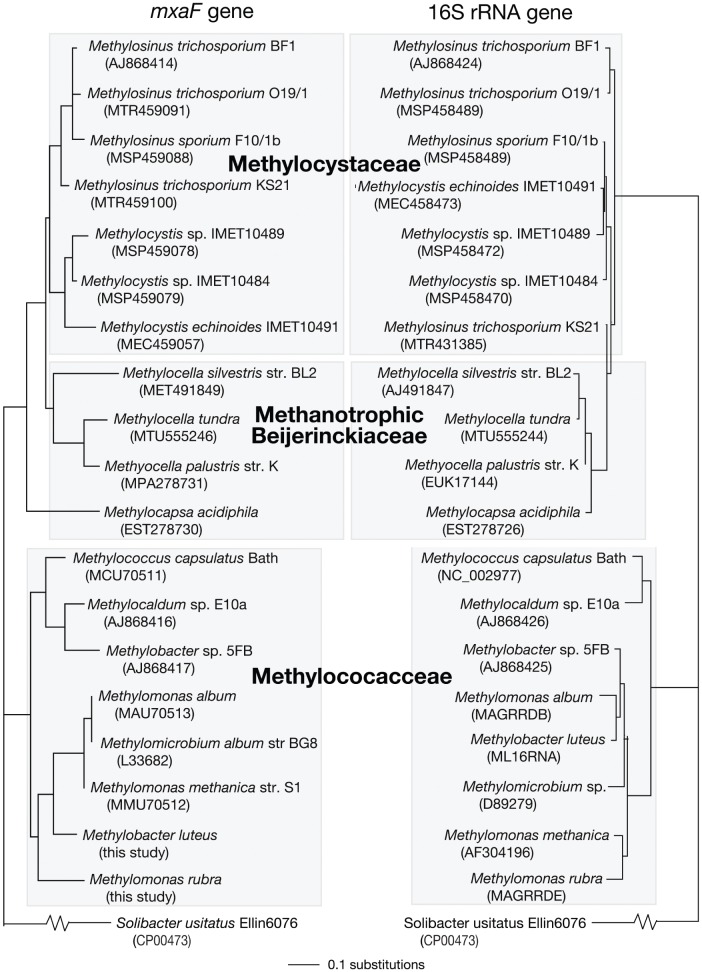
Congruency tests between *mxaF* and 16S rRNA gene nucleotide sequences of methanotrophs from GenBank database. Phylogenetic trees for congruency tests based on maximum likelihood (ML) analysis of *mxaF* (∼513 bp) and 16S rRNA gene nucleotide sequences (∼1471 bp) from methanotrophs in GenBank, including the *mxaF* nucleotide sequences of *Methylomonas rubra* and *Methylobacter luteus* sequenced in this study. The ADH gene of *Solibacter usitatus* Ellin 6076 was used as outgroup. Methanotrophs (in the Methylococcaceae, Methylocystaceae and Beijerinckiaceae) are indicated by shaded clusters. Accession numbers of *mxaF* and 16S rRNA gene sequences downloaded from GenBank are indicated in parentheses. Bootstrap values from 1,000 replicates are indicated at the nodes of branches (if >50). The scale bar represents the number of nucleotide changes.

Congruency tests showed that the *mxaF* and 16S rRNA genes of representative methanotroph species do not share congruent tree topologies. Topology tests failed to reject the null hypothesis that *mxaF* and 16S rRNA gene trees, when each was constrained to match the topology of the other gene, have significantly different likelihood scores. The SH test examines the topology, but does not indicate specific nodes or taxa causing incongruence [47]. Trees for *mxaF* gene, 16S rRNA gene, and combined *mxaF*+16S rRNA genes were observed to be incongruent, with incongruencies for representative taxa from the Methylocystaceae and Methylococcaceae, and the methanotrophic Beijerinckiaceae (Fig. 1).

### mxaF Gene Phylogeny

**Figure 2:**
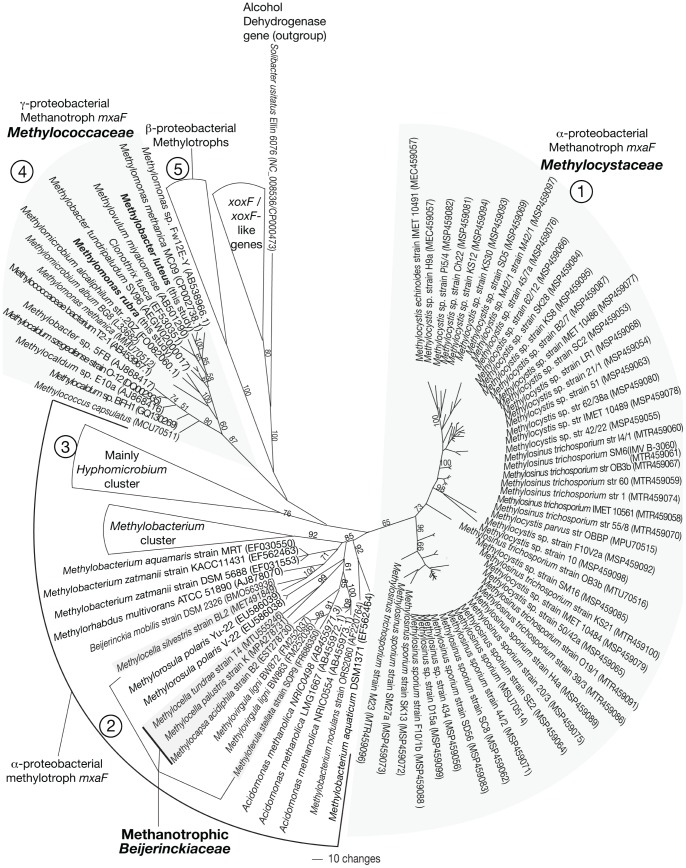
Simplified phylogenetic tree of methanotrophs and their close relatives based on *mxaF* nucleotide sequences from GenBank database. Unrooted phylogenetic tree based on maximum parsimony (MP) analysis of known proteobacterial partial *mxaF* and *xoxF*/*xoxF*-like nucleotide sequences (∼513 bp) from GenBank and the *mxaF* nucleotide sequences (in bold) of *Methylomonas rubra* and *Methylobacter luteus* sequenced in this study. The ADH gene of *Solibacter usitatus* Ellin 6076 was used as outgroup. Accession numbers of sequences downloaded from GenBank are indicated in parentheses. Bootstrap values from 1,000 replicates are indicated at the nodes of branches (if >50). The three bacterial families containing methanotrophs (Methylococcaceae, Methylocystaceae and methanotrophic members of the Beijerinckiaceae) are indicated by shaded clusters and the other alphaproteobacterial and betaproteobacterial methylotrophs are delineated by lines. The identity of *mxaF* and *mxaF*-like sequences from the “*Methylobacterium* cluster (within cluster 2)”, “Mainly *Hyphomicrobium* (Cluster 3)”, “β-proteobacterial methylotrophs (Cluster 5)”, and “*xoxF/xoxF*-like genes” is shown in Table 2. The scale bar represents the number of nucleotide changes. The complete phylogenetic tree of methanotrophs and their close relatives based on *mxaF* nucleotide sequences is shown in Supplement S1.

The simplified *mxaF* gene tree (based on all codon positions) for known proteobacterial methylotrophs is shown in Fig. 2. Trees resulting from analyses of 1^st^ and 2^nd^ codon positions and inferred *mxaF* amino acid sequences of methylotrophs were identical in branching order of major taxa (not shown). Figure 2 indicates five distinct clusters of methylotrophs: (a) cluster 1 encompassing the Methylocystaceae, (b) cluster 2 containing mostly methylotrophic *Methylobacterium* sp. and methanotrophic Beijerinckiaceae (*Methylocapsa* and *Methylocella*), (c) cluster 3 containing mostly *Hyphomicrobium* sp., (d) cluster 4 consisting solely of the monophyletic family Methylococcaceae, including sequences obtained in this study for the cultured members *Methylomonas rubra* and *Methylobacter luteus*, and (e) cluster 5 containing betaproteobacterial methylotroph genera. In addition, a well-supported monophyletic cluster 6 falls outside of other *mxaF* clusters and consists of *xoxF* gene sequences, which are distantly related to methylotroph *mxaF* sequences [32]. The list of taxa comprising the non-methanotrophic methylotrophs and *xoxF*/*xoxF*-like clusters in Fig. 2 is shown in Table 2. The complete tree with all taxa (n = 145) is shown in Supplement S1.

**Table 2:** Taxa included (but not shown) in the phylogenetic analyses for Fig. 2.

Cluster name	Taxa and GenBank Accession number (in parentheses)
*Methylobacterium* cluster(within cluster 2)	*Methylobacterium nodulans* strain ORS2060 (AF220764)*Methylobacterium aquaticum* DSM1371 (EF562464)*Methylobacterium* sp. MP3 (EF030549)*Methylobacterium lusitanum* strain MP2 (EF030548)*Methylobacterium rhodesianum* strain DSM5687 (EF562473)*Methylobacterium hispanicum* strain DSM16372 (EF562468)M*ethylobacterium jeotgali* strain S2R03-9 (EF031552)*Methylobacterium* sp. MPS (EU047511)*Methylobacterium mesophilicum* strain DSM1708 (EF562470)*Methylobacterium organophilum* DSM760 (EF562471)*Methylobacterium radiotolerans* JCM2831 1819 (EF562472)*Methylobacterium fujisawaense* strain KACC10744 (EF562467)*Methylobacterium oryzae* strain CBMB20 (EF562478)*Methylobacterium fujisawaense* strain MP1 (EF030547)*Methylobacterium oryzae* strain CBMB110 (EF562476)*Methylobacterium suomiense* strain KCTC12963 (EF562474)*Methylobacterium suomiense* strain CBMB130 (EF562479)*Methylobacterium suomiense* strain CBMB120 (EF562477)*Methylobacterium thiocyanatum* strain DSM11490 (EF562475)*Afipia felis* strain 25E-1 (AY8488 26)*Afipia felis* strain RD1 (AY848827)*Methylobacterium podarium* strain FM4 (AY468366)*Methylobacterium extorquens* strain DSM1337 (EF562466)*Methylobacterium dichloromethanicum* strain DSM6343 (AJ878068)*Methylobacterium dichloromethanicum* strain KACC11438 (EF562465)*Methylobacterium rhodinum* strain DSM2163 (EF562487)
Mainly *Hyphomicrobium*(Cluster 3)	*Hyphomicrobium* sp. strain B 314 (HSMXAF314)*Hyphomicrobium* sp. strain B 327 (HSMXAF327)*Hyphomicrobium* sp. strain DPB 2c (HSMXAF2C)*Hyphomicrobium aestuarii* (HAMXAF)*Hyphomicrobium* sp. strain B 583 (HSMXAF583)*Hyphomicrobium* sp. strain B 520 (HSMXAF520)*Hyphomicrobium* sp. strain B 294 (HSMXAF294)*Hyphomicrobium* sp. strain P 324 (HSMXAF324)*Hyphomicrobium zavarzinii* (HZMXAF)*Hyphomicrobium* sp. strain B 69 (HSMXAF69)*Hyphomicrobium vulgare* (HVMXAF)*Hyphomicrobium* sp. strain P 768 (HSMXAF768)*Hyphomicrobium* sp. strain P 495 (HSMXAF495 )Hyphomicrobium methylovorum (AM004097)*Hyphomicrobium* sp. P 495 (HSMXAF495)*Hyphomicrobium* sp. P 768 (HSMXAF768)*Hansschlegelia plantiphila* strain S1 (DQ652143)*Hansschlegelia plantiphila* strain S2 (DQ652144)*Hansschlegelia plantiphila* strain S4 (DQ652145)*Paracoccus kondratievae* NCIMB 13773 (AJ878072)*Methylosulfonomonas methylovora* (MMU70525)*Ancylobacter aquaticus* strain IAM 12364 (AB455976)*Albibacter methylovorans* DSM 13819 (AJ878069)*Methylopila capsulata* ATCC 700716 (AJ878071)
β-proteobacterial Methylotrophs(Cluster 5)	*Flavisobacter* sp. Vu-144 (EU912489)*Duganella* sp. B41 (EU439303)*Methylophilus methylotrophus* (MMU41040)*Methylobacillus glycogens* ATCC29475 (AJ878073)*Methylovorus* sp. SS1 (AF184915)
*xoxF/xoxF*-like genes	*Paracoccus denitrificans* (U34346)*Rhodobacter sphaeroides* 2.4.1 (CP000143)*Methylobacterium radiotolerans* JCM 2831 Mrad 2831 1932 (NC_010505)*Methyloversatilis universalis* strain FAM5 (EU548068)Burkholderiales bacterium RZ18-153 (EU548065)*Methylobacterium extorquens* PA1 Mext_1809 (NC_010172)*Methylobacterium chloromethanicum* CM4 Mchl2145 (NC_011757)*Methylobacterium extorquens* AM1 MexAM1 META1p1740 (NC_012808)*Methylobacterium* sp. 4–46 M446 5752 (NC_010511)*Methylobacterium radiotolerans* JCM 2831 Mrad 2831 0508 (NC_010505)*Methylobacterium* sp. 4–46 M446 2082 (NC_010511)

Cluster 1 includes all members of the Methylocystaceae, forming two separate sub-clusters. Both sub-clusters are polyphyletic for the two genera *Methylosinus* and *Methylocystis*. The methanotrophic Beijerinckiaceae (genera *Methylocapsa*, *Methylocella* and *Methyloferula*) are placed polyphyletically within cluster 2 with other methylotrophs (Fig. 2). There is a lack of distinction in the placement of species of methanotrophic Beijerinckiaceae in cluster 2: *Methylocapsa* is most closely related to the non-methanotrophic genus *Acidomonas* [48], while the close relatives *Methylocella palustris* strain K and *Methylocella tundrae* cluster with the methylotrophic *Methylorosula polaris*, gen. *nov*. Yu-22 and V-22 [49], *Methylocella silvestris* clusters with the non-methanotrophs, *Beijerinckia mobilis* DSM 2326, and *Methyloferula stellata* clusters with the methylotrophic *Methylovirgula ligni* strains BW863 and BW872 [50]. Based on the *mxaF* gene tree (Fig. 2), the alphaproteobacterial groups – the Methylocystaceae (cluster 1), alphaproteobacterial methylotrophs including the methanotrophic Beijerinckiaceae (clusters 2 and 3) – are more closely related to one another than to betaproteobacterial methylotrophs (cluster 5) and gammaproteobacterial methanotrophs (cluster 4). This pattern is also reflected in the 16S rDNA phylogeny.

Pairwise nucleotide sequence variations within the families Methylocystaceae and Methylococcaceae and between methanotrophic Beijerinckiaceae were ≥87%, ≥75%, and ≥78%, respectively. In contrast, the *xoxF* cluster shared ≤69% identity with all *mxaF* genes, and *Solibacter usitatus* Ellin 6076 ADH (outgroup) shared ≤55% identity with all *mxaF* genes.

### Phylogenetic Analyses of PCR-amplified Environmental mxaF Gene Sequences

**Figure 3:**
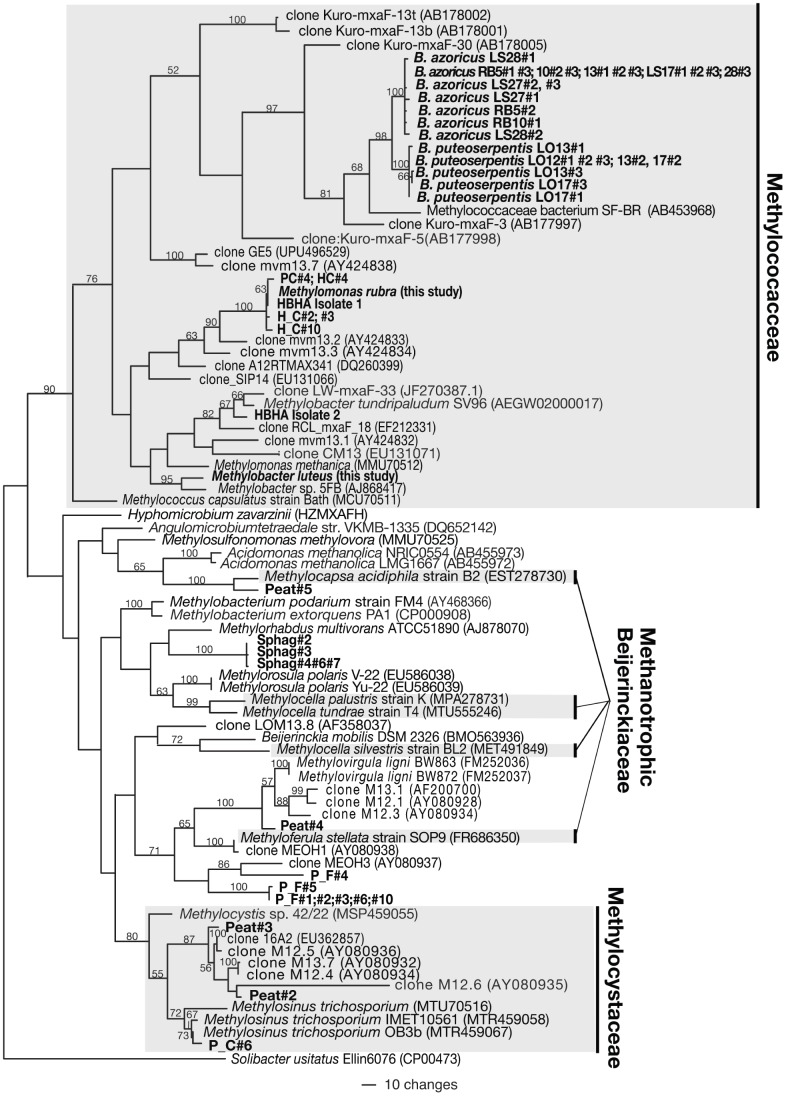
Phylogenetic tree of environmental *mxaF* gene sequences detected in this study. Phylogenetic tree based on MP analyses of environmental partial *mxaF* nucleotide sequences (∼513 bp) detected in this study (in bold) in comparison with their close relatives, with *Solibacter usitatus* Ellin6076 as outgroup. All *mxaF* sequences were obtained using primer pair F1003 and R1561, except for the 13 putative symbiont *mxaF* genes from *Bathymodiolus azoricus* and *B. puteoserpentis,* which were obtained using primer pair F1003degen and R1561degen. Accession numbers of sequences downloaded from GenBank are indicated in parentheses. Bootstrap values from 1,000 replicates are indicated at the nodes of branches (if >50). Clone sequences are labeled P_C, pine soil (control); P_F, pine soil (fertilized); H_F, hardwood soil (fertilized); Sphag, *Sphagnum* moss; HBHA, Halls Brook Holding Area; RB, Rainbow; LS, Lucky Strike; LO, Logatchev, followed by clone number (#).Methanotrophs found in the three bacterial families (Methylococcaceae, Methylcystaceae and Beijerinckiaceae) are shaded. Bootstrap values from 1,000 replicates are indicated at the nodes of branches (if >50). The scale bar represents the number of nucleotide changes.

All inferred amino acid sequences translated from *mxaF* genes amplified in this study possessed the three amino acid residues involved in the interactions of the active site. MP phylogenetic trees based on all nucleotides, 1^st^ and 2^nd^ codon positions, and *mxaF* amino acid sequences were identical in topology; the tree based on all three nucleotides is shown in Fig. 3. Of the *mxaF* clones amplified from Harvard Forest soils (labeled P_C, P_F, and H_F), one was placed in the Methylocystaceae and five were placed in the Methylococcaceae, with the remaining three (P_F#1;#2;#3;#6;#10, P_F#4, and P_F#5) closely related to environmental clones detected in other soils. Three sequences detected in *Sphagnum* moss (labeled Sphag, Fig. 3) clustered with the methylotroph *Methylorhabdus multivorans,* although this clade lacked bootstrap support. Of the four sequences detected in peat (labeled Peat) in the northern WI bog, one (Peat#4) clustered with *mxaF* sequences from clones detected in acidic forest and acidic peatland soils, and one was most close-related to *Methylocapsa acidiphila* str. BL2, isolated from acidic peat in Siberia [51]. Both of the lake isolates were placed in the Methylococcaceae, with HBHA isolate 1 most closely related to *Methylomonas rubra* and HBHA isolate 2 related to *Methylobacter tundripaludum* SV96 and clone LW-mxaF-33 (Fig. 3).

For the bathymodiolid mussels, three clones per gill were sequenced from a total of nine individuals (three per site) representing two host species: *B. azoricus* [at Lucky Strike (LS) and Rainbow (RB) vent sites] and *B. puteoserpentis* (at Logatchev (LO) vent site] (27 total clones sequenced). Seven unique sequences were detected among the 18 *B. azoricus* clones, and five unique sequences were detected among 9 *B. puteoserpentis* clones. Of the 7 unique sequences detected in *B. azoricus*, 2 were from RB vent site, 4 were from LS vent site, and one was from both RB and LS sites (Fig. 3). These 12 unique sequences vary from one another at up to 27 synonymous nucleotide positions (coding for the same amino acid residues) and 6 nonsynonymous nucleotide positions (coding for different amino acid residues) (see Supplement S1 for details). These *mxaF* gene sequences, which possessed all three amino acid residues involved in the active site of the enzyme, were amplified from all of the mussel specimens. In congruence with 16S rRNA gene phylogenies for the methanotrophic *Bathymodiolus* endosymbionts, the mussel *mxaF* sequences clustered within the Methylococacceae. The tight clustering of these *mxaF* sequences of two different *Bathymodiolus* species from sites >2600 km apart, argues that these were amplified from the symbionts. Thus, we refer to the origin of the sequences as “putative” symbiont *mxaF* sequences. Most mussels (except *B. azoricus* LS13, *B. azoricus* LS17, and *B. puteoserpentis* LO12) possessed more than one unique *mxaF* sequence. While we cannot exclude the possibility of PCR error as the cause of sequence variance, mussel-derived *mxaF* sequences formed two closely related but independent monophyletic clades, separated according to host species, with the *B. azoricus* cluster from LS+RB sharing ≥99% nucleotide identity and the cluster from *B. puteoserpentis* (at LO) sharing ≥98% nucleotide identity. Nucleotide identity between sequences from both clusters is 95–96%. All 12 unique putative endosymbiont *mxaF* gene sequences are most closely related (87–88% nucleotide identity) to a free-living methanotrophic bacterium, SF-BR, isolated from San Francisco Bay, CA [52] (Fig. 3). When nucleotide variations were compared with the inferred amino acid residues, 9 of the *mxaF* gene sequences varied from one another at up to 27 synonymous sites and coded for the same inferred amino acid sequence. The remaining 3 sequences LS27#1, LS28#2, and RB10#1, possessed the 20 or more synonymous sites identical with sequences from RB and LS, but possessed, additionally, 1–3 nonsynonymous changes, coding for up to 3 other unique amino acid sequences (see Supplement S1). Our analysis of *mxaF* genes suggests that the putative endosymbionts of *B. azoricus* and *B. puteoserpentis* arose from a single free-living ancestor in the Methylococcaceae. Calculated Ka/Ks (ratio of rate of nonsynonymous substitutions to synonymous substitutions) values for all Bathymodiolid putative endosymbionts, all free-living members of Methylococcaceae and between all putative Bathymodiolid endosymbionts compared to members of Methylococcaceae, averaged 2.0×10^−2^, and did not differ significantly among these groups (*P*>0.05, unpaired one-tailed *t*-test+ANOVA) (data not shown). These uniformly low Ka/Ks values among all members of the Methylococcaceae (whether putatively symbiotic or free-living) indicate that most nucleotide substitutions are synonymous (see Supplement S1). If random errors in nucleotide incorporation during PCR had occurred, calculated Ka/Ks values for *mxaF* genes of the putative endosymbionts would likely have been significantly higher (i.e., approaching 1).

## Discussion

Establishing the link between microbial phylogeny and physiology is complicated by the high level of physiological diversity in most microbial taxa (e.g., many microbes utilizing several carbon sources), and the potential for horizontal gene transfers – the movement of microbial genes between divergent genomes. Consequently, it is necessary to evaluate the accuracy of candidate functional genes as a diagnostic of key metabolic processes, as well as being accurate markers of evolutionary history. Doing so requires evaluating the result of phylogenetic reconstruction of gene sequences from all cultured organisms possessing the gene, before analyses of sequences from uncultured environmental clones. Methanotrophs are unique due to their preference for methane as a metabolic substrate and their possession of functional genes involved in this process. Here, we investigated the phylogeny, based on the *mxaF* gene, of all known proteobacterial methanotrophs from GenBank database and highlighted the *mxaF* gene’s ability to detect most methanotrophic bacteria and describe their molecular diversity in natural environments.

### mxaF and 16S rRNA Gene Phylogenies

In this study, the topological differences between 16S rRNA and *mxaF* gene trees, as seen in incongruencies between both trees (Fig. 1), suggest multiple occurrences of horizontal gene transfer in the *mxaF* genes of many methanotrophic taxa in the Methylococcaceae, Methylocystaceae, and Beijerinckiacea. Phylogenetic analyses based on *mxaF* genes placed the vast majority of cultivated proteobacterial methanotrophs, excluding members of the genera *Methylocapsa*, *Methylocella*, and *Methyloferula*, in distinct and coherent clades representing the Methylococcaceae and Methylocystaceae, with higher nucleotide identity between taxa within these clades than between members of different clades. These family-level clade distinctions are consistent with patterns evident in the 16S rDNA phylogeny. However, below the family level, *mxaF*-based analyses failed to differentiate between distinct subclades based on genera.

The *mxaF* gene has poor resolving power for methanotrophs within the Beijerinckiaceae. Our *mxaF* gene tree (Fig. 2) indicates that these methanotrophs, composed of the genera *Methylocapsa*, *Methylocella*, and *Methyloferula*, are polyphyletic, sharing common ancestry with other alphaproteobacterial methylotrophs. This pattern corroborates previous studies, which assessed fewer methylotroph taxa [23], [49], [53], and showed different topologies between *mxaF* and 16S rRNA genes, for example, in the genus *Methylocella* [22], [50], [53]. The more extensive *mxaF* gene phylogeny inferred here (compared to previous studies) suggests that methanotrophy (a) arose once in the Beijerinckiaceae and was lost by some methylotrophic taxa, (b) arose separately in more than one taxon in the Beijerinckiaceae, and/or (c) multiple occurrences of horizontal gene transfers have occurred in the common ancestor of methanotrophic Beijerinckiaceae. It therefore is difficult to ascertain whether microorganisms with *mxaF* gene sequences placed near the polyphyletic Beijerinckiaceae genera *Methylocapsa, Methylocella*, and *Methyloferula* are indeed methanotrophic.

Overall, our data suggest that the partial *mxaF* gene (∼550 bp) amplified by the primer set mentioned is a useful phylogenetic marker and provides sufficient resolution to broadly discriminate between known proteobacterial methanotroph families via the Methylococcaceae and Methylocystaceae clusters, which together encompass the vast majority of known methanotrophs. However, it has poor resolution at the sub-family level across the Methylococcaceae (not shown in previous studies) and Methylocystaceae, and ambiguities exist between the methanotrophic Beijerinckiaceae and other methylotrophs in the Alphaproteobacteria, due possibly to horizontal transfers of the *mxaF* gene.

### mxaF Sequences from Diverse Environments, Isolates and Endosymbiotic Methanotrophs

The *mxaF* gene was used here to examine the phylogenetic placement and diversity of over fifty novel sequences of putative methanotrophs from a range of natural environments and from cultured isolates in light of evolutionary information from the *mxaF* gene phylogeny determined above. The *mxaF* datasets from Harvard Forest soil, peat and *Sphagnum* moss from northern Wisconsin, and the HBHA water column contained diverse sequences that clustered primarily in the Methylococcaceae and Methylocystaceae, as well as sequences related to methanotrophic Beijerinckiaceae and other alphaproteobacterial methylotrophs. Notably, the sample of *Sphagnum* moss contained three sequences (Sphag#1, Sphag#2, and Sphag#3), suggestive of three strains, most closely related to the methylotroph *Methylorhabdus multivorans*. However, these three sequences are also closely related to the methanotrophic Beijerinckiaceae genus *Methylocella* (Fig. 3). Given the uncertainty with which *mxaF* discriminates between methanotrophic Beijerinckiaceae and certain alphaproteobacterial methylotrophs discussed above, the sequences detected in peat, forest soils and *Sphagnum* moss could represent methanotrophic bacteria.

Our analyses also provide the first insights into the phylogenetic placement and biogeography of the previously unknown *mxaF* genes from putative methanotrophic endosymbionts of deep-sea hydrothermal vent Bathymodiolid mussels. *Bathymodiolus azoricus* and *B. puteoserpentis* are the dominant species of mussels within the two spatially separate mussel hybrid zones of Lucky Strike and Rainbow, and Logatchev on the Mid-Atlantic Ridge [54]. Here, dual bacterial endosymbionts provide nutrition to the mussel hosts through thiotrophy and methanotrophy, but the mode of methanotrophic symbiont transmission, whether vertical (symbionts are passed from parent to offspring) or horizontal (symbionts are taken up from the environment, or from co-occurring hosts) is not known [38], [39], [55], [56]. Here, we show that these novel *mxaF* gene sequences of putative endosymbiotic methanotrophs belong to the Methylococcaceae, in agreement with their placement in the Gammaproteobacteria with other methanotrophs based on 16S rDNA phylogeny [6]. *B. azoricus* mussels at the adjacent Lucky Strike and Rainbow vent sites harbored closely related putative methanotrophic symbionts, with most mussels possessing a heterogeneous population of putative endosymbionts, based on the closely-related but unique *mxaF* gene sequences we detected. This result is consistent with the hypothesis of environmental acquisition of mussel symbionts [38], [55], [57], [58], where each mussel may be expected to contain multiple genetic variants.

In contrast, *B. puteoserpentis* mussels at Logatchev, a vent site over 2600 km away, harbored a separate monophyletic group of closely related putative methanotrophic symbionts. The phylogenetic clustering of *B. azoricus* and *B. puteoserpentis* symbionts in two distinct clades suggests either taxon-specific differences in the specificity of the symbiont-host relationship (i.e., each host species associates with a unique symbiont strain) or that *B. azoricus* mussels at Lucky Strike and Rainbow acquire putative symbionts from an environmental pool that is genetically distinct from that available to *B. puteoserpentis* mussels at Logatchev vent site. Our phylogenetic analyses of *mxaF* gene sequences indicate that the putative endosymbiotic methanotrophs have diverged from an ancestral sequence into two monophyletic groups, either in response to host-symbiont co-speciation or to geographic separation. Indeed, the fragmented distribution of deep-sea hydrothermal vents may promote spatial isolation that acts as a barrier to symbiont dispersal [59]. With more information, the extent to which symbiont diversification is driven by either geographic isolation and symbiont-host specificity and co-evolution, can be assessed.

Proteobacterial methanotrophs currently constitute the vast majority of known aerobic methane oxidizing bacteria. In this study, we demonstrate the usefulness of the *mxaF* gene in studying proteobacterial methanotroph diversity in non-anoxic environments. Studying the evolutionary history of this gene from known methanotrophs extensively may provide insights into the placement of novel taxa detected in different environments and avoid incorrect inferences from their phylogenetic placement. Our results indicate the *mxaF* gene can be a functional and phylogenetic marker for proteobacterial methanotrophs, providing more information about an important group of microorganisms involved in the global biogeochemical methane cycle.

## Supporting Information

Supplement S1 (DOCX)
