# Supplementary material for: The Methanol Dehydrogenase Gene, mxaF, as a Functional and Phylogenetic Marker for Proteobacterial Methanotrophs in Natural Environments
Source: PLoS One. 2013 Feb 22;8(2):e56993. doi: 10.1371/journal.pone.0056993 (PMC3579938; doi:10.1371/journal.pone.0056993)

**Supplement S1 Supporting Information**

**Title: The Methanol Dehydrogenase Gene, *mxaF*, as a Functional and Phylogenetic Marker for Proteobacterial Methanotrophs in Natural Environments**

**Authors and Affiliations:**

Evan Lau^1, 2^, Meredith C. Fisher^2^, Paul A. Steudler^3^, and Colleen M. Cavanaugh^2^

^1^ Department of Natural Sciences and Mathematics, West Liberty University, West liberty, WV, USA

^2^ Department of Organismic and Evolutionary Biology, Harvard University, Cambridge, MA, USA

^3^ The Ecosystems Center, Marine Biological Laboratory, Woods Hole, MA, USA

Corresponding Author: Evan Lau, West Liberty University, Department of Mathematics and Natural Sciences, 208 University Drive, CUB #139, evanlau28@gmail.com, (304)336-8529

**Methods Supplement**

**Ka/Ks calculations.** Nucleotide data from known members of the Methylococcaceae and *mxaF* clone sequences found in this study were aligned (in correct reading frame) using MacClade, based on their translated amino acid sequences verified with sequences downloaded from GenBank. The number of amino acid synonymous substitutions (Ks), non-synonymous substitutions (Ka), and Ka/Ks values between species was estimated and automatically corrected using the MYN approximation methods on the KaKs_Calculator (<http://code.google.com/p/kaks-calculator/wiki/KaKs_Calculator>) [1]. The *mxaF* gene sequence for *Methylococcus capsulatus* was selected as reference sequence when calculating Ka, Ks and Ka/Ks, as *M. capsulatus* was placed at the base of the *mxaF* gene tree for the Methylococcaceae. Unpaired one-tailed unequal variances *t*-test, available on Excel^®^ ver. 12.3.2 (Microsoft^®^ Corp.) was used to compare the mean Ka/Ks values of the three groups to determine whether they are significantly different. The null hypothesis for each test: the calculated *t*-value does not exceed critical *t*-value (for *P*=0.95).

**Results supplement**

In addition to the uniformly low Ka/Ks values among all members of the Methylococcaceae, the likelihood that some of the putative symbiont *mxaF* sequences may be the result of PCR error is further reduced from the following observations: (a) none of the putative symbiont *mxaF* gene sequences contained a nonsense substitution or termination codon (which would lead to an abnormally-shortened and likely non-functional MDH), (b) these putative symbiont *mxaF* sequences possessed all three amino acid residues involved in the interactions of the active site, and (c) most of the *mxaF* nucleotide differences (for 9 out of 12 unique sequences) were located at synonymous sites.

**References supplement**

1. Zhang Z, Li J, Zhao X-Q, Wang J, Wong GK-S, et al. (2006) KaKs calculator: calculating Ka and Ks through model selection and model averaging. Genom Prot Bioinfo 4: 259-263.

**Figure Legends supplement**

**Fig. S1.** Complete phylogenetic tree of methanotrophs and their close relatives based on *mxaF* nucleotide sequences from GenBank database. Unrooted phylogenetic tree based on maximum parsimony (MP) analysis of known proteobacterial partial *mxaF* and *xoxF*/*xoxF*-like nucleotide sequences (~513 bp) from GenBank and the *mxaF* nucleotide sequences (in bold) of *Methylomonas rubra* and *Methylobacter luteus* sequenced in this study. The ADH gene of *Solibacter usitatus* Ellin 6076 was used as outgroup. Accession numbers of sequences downloaded from GenBank are indicated in parentheses. Bootstrap values from 1,000 replicates are indicated at the nodes of branches (if >50). The three bacterial families containing methanotrophs (Methylococcaceae, Methylocystaceae and methanotrophic members of the Beijerinckiaceae) are indicated by shaded clusters and the other alphaproteobacterial and betaproteobacterial methylotrophs are delineated by lines. The scale bar represents the number of nucleotide changes.

**Fig. S2.** Alignment of 12 unique *mxaF* gene sequences from *B. azoricus* and *B. puteoserpenti*s from Lucky Strike, Rainbow and Logatchev vent sites, with the inferred amino acid sequence common to 9 of them. Synonymous (S) and nonsynonymous (N) nucleotide substitutions are shaded in green and red boxes, respectively.

**Fig. S1.**


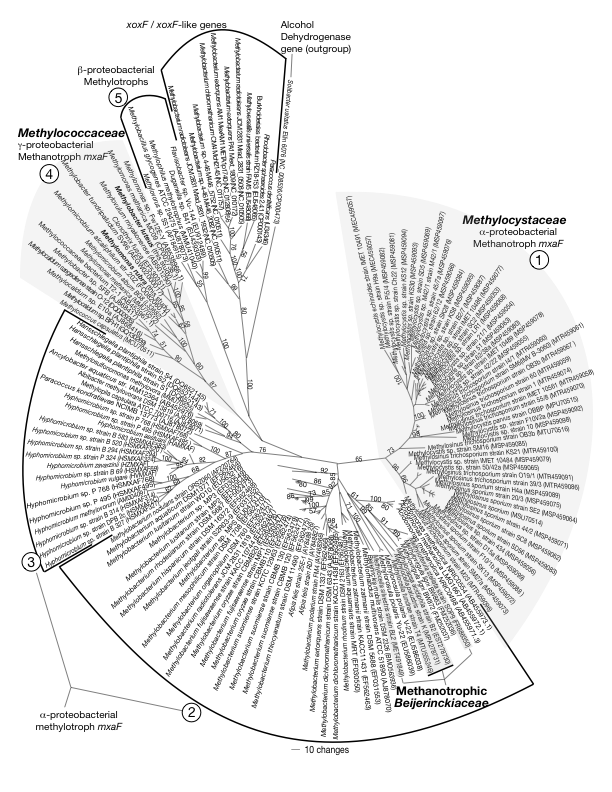


**Fig. S2.**


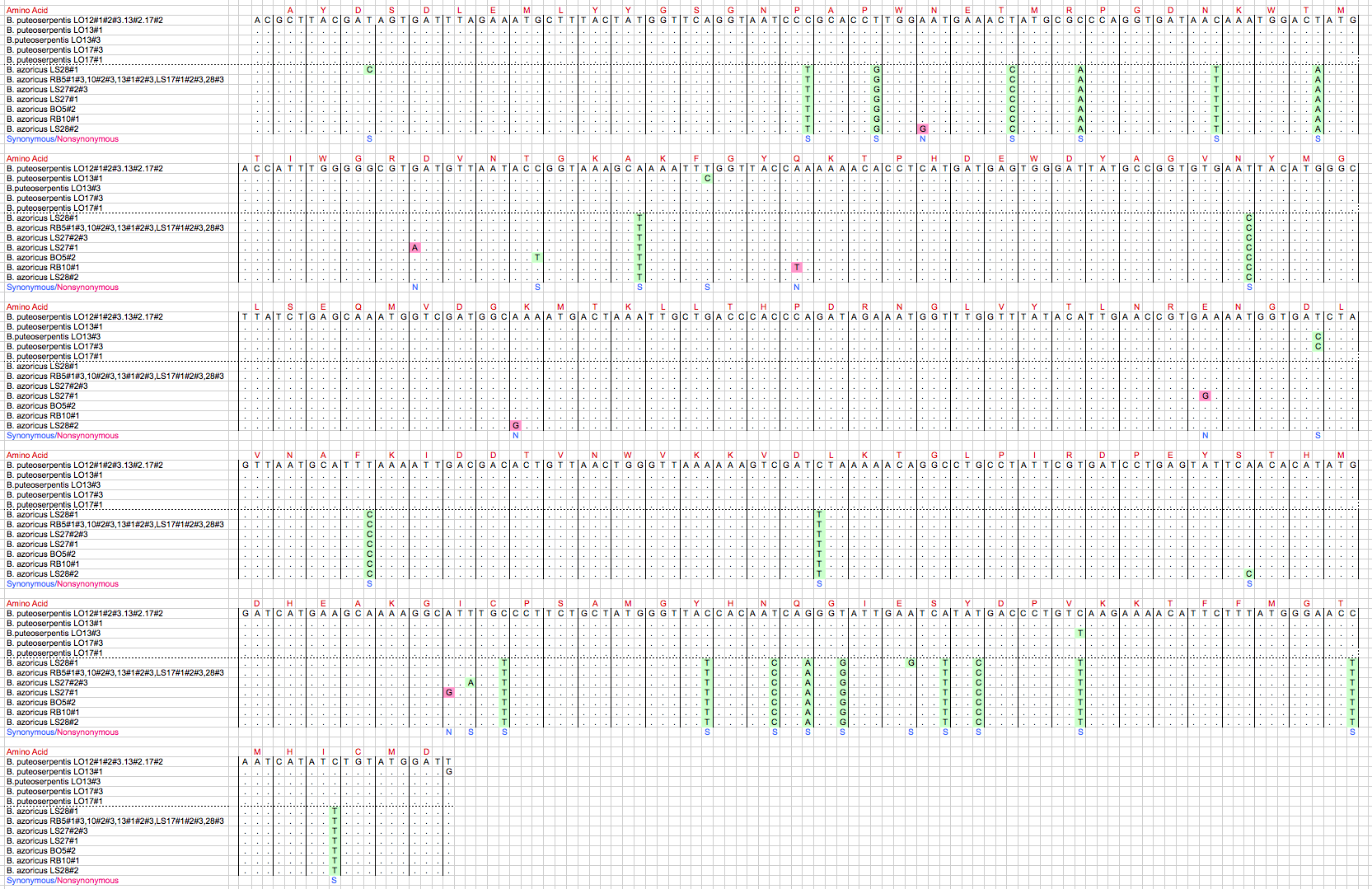

Supplement: Supplement S1 — (DOCX) [file pone.0056993.s001.docx]
